# Expanding 3D Nanoprinting Performance by Blurring the Electron Beam

**DOI:** 10.3390/mi12020115

**Published:** 2021-01-22

**Authors:** Lukas Matthias Seewald, Robert Winkler, Gerald Kothleitner, Harald Plank

**Affiliations:** 1 Christian Doppler Laboratory for Direct-Write Fabrication of 3D Nano-Probes, Institute of Electron Microscopy and Nanoanalysis, Graz University of Technology, 8010 Graz, Austria; lukas.seewald@felmi-zfe.at (L.M.S.); robert.winkler@felmi-zfe.at (R.W.); 2 Graz Centre for Electron Microscopy, Steyrergasse 17, 8010 Graz, Austria; gerald.kothleitner@felmi-zfe.at; 3 Institute of Electron Microscopy and Nanoanalysis, Graz University of Technology, 8010 Graz, Austria

**Keywords:** 3D-nanoprinting, additive manufacturing, direct-write manufacturing, metallic nanostructures, helices, nanowires, focused electron beam induced deposition, platinum

## Abstract

Additive, direct-write manufacturing via a focused electron beam has evolved into a reliable 3D nanoprinting technology in recent years. Aside from low demands on substrate materials and surface morphologies, this technology allows the fabrication of freestanding, 3D architectures with feature sizes down to the sub-20 nm range. While indispensably needed for some concepts (e.g., 3D nano-plasmonics), the final applications can also be limited due to low mechanical rigidity, and thermal- or electric conductivities. To optimize these properties, without changing the overall 3D architecture, a controlled method for tuning individual branch diameters is desirable. Following this motivation, here, we introduce on-purpose beam blurring for controlled upward scaling and study the behavior at different inclination angles. The study reveals a massive boost in growth efficiencies up to a factor of five and the strong delay of unwanted proximal growth. In doing so, this work expands the design flexibility of this technology.

## 1. Introduction

Focused electron beam induced deposition (FEBID) is a mask-less direct-write manufacturing technology, where surface adsorbed precursor molecules are dissociated, and thereby immobilized, upon irradiation with a focused electron beam [1]. Aside from the additive character with minimal demands on the substrate materials and morphologies, this technology allows for the fabrication of freestanding, 3D architectures with feature sizes down to the sub-20 nm range [2,3]. As FEBID based 3D nanoprinting [4] (3D-FEBID) is realized by controlled lateral movement of the electron beam, the design flexibility is very high, which opens up entirely new possibilities for, e.g., artificial magnetic [5,6] and plasmonic [7,8,9] structures and advanced scanning probe microscopy tips [10].

To expand the pool of potential applications, this work aims to explore the fabrication of open helices with helical cylinder diameters in the range of half a micrometer. Although discussions regarding the FEBID fabrication of helices for use as optical metamaterials [9]—or to study fundamental aspects (e.g., magnetism) at the nanoscale [6]—are present in the literature, such mentioned helices either have a comparably smaller helical cylinder diameter and/or they are tightly packed. The increased helical diameter poses a significant challenge to 3D-FEBID fabrication as the length of the helical wire increases with the helix diameter and the inclination angle, resulting in very long effective segment lengths of several micrometers with diameters in the sub-100 nm range. Although the latter is a clear strength of 3D-FEBID and beneficial for some applications, it entails certain challenges. (i) The narrow nanowires are mechanically fragile [11] and easily bend upon further direct or proximal irradiation by the electron beam [12]. Since segment widths depend on the actual inclination angle [3], this is of particular interest for shallow inclined segments. (ii) Due to the low thermal conductivity of many as-deposited FEBID materials in combination with the small cross sections, the problem of electron beam induced heating (EBIH) becomes relevant [13]. Heat brought into the structure by inelastic scattering events of the electron beam cannot be sufficiently dissipated, which imposes a thermal gradient and gradually increases the local temperature at the beam impact region. In turn, gas dynamics are radically altered, which leads to decaying and eventually collapsing growth behavior. Compensation strategies have been proposed to stabilize the growth to a certain extent [14], which, however, requires simulation know-how. (iii) Another challenge for 3D-FEBID is the occurrence of co-deposition (also termed secondary deposition [15], parallel growth and branching [16]), which results in the formation of unintended and mostly unwanted deposits beneath the actual deposits. Apparently, structural fidelity is put at stake upon formation of co-deposits, and therefore, must be avoided at all cost. Experiments and simulations suggest that the formation of a co-deposit is driven by transmitted electrons [15,17], however, the topic of the occurrence of co-deposition is incompletely understood. (iv) Furthermore, the volume growth rates and efficiencies obtained with 3D-FEBID are meager compared to other fabrication techniques [18]. The main reason for the poor growth rates lies in the high current density entailed in the nanometer sized electron beam, which shifts the growth quickly to an adsorption–desorption balanced regime [19]. In this context, Plank et al. showed that the volume growth efficiency of FEBID can be increased upon introduction of a defocus to the electron beam [20]. As a consequence of the wider beam, the fabricated pillars grew at wider dimensions. In more recent studies, Pablo-Navarro et al. and Kuhness et al. showed that the diameter of a vertical pillar structure can be modulated by means of the dynamic adjustment of the beam diameter during deposition [21,22]. Addressing 3D-FEBID in particular, Winkler et al. employed a defocused electron beam for the growth of so called diving boards. Upon introduction of a slightly defocused electron beam, segments grew at a decreased inclination angle with increased wire diameter. They, however, used the results to highlight the importance of a carefully focused electron beam as a prerequisite for predictable and reliable fabrication [23].

In the context of the aforementioned main issues during 3D-FEBID fabrication of long segments in helical designs, an *increased wire diameter* by the *introduction of a beam defocus* should be beneficial in several aspects. First and most obvious, the mechanical stability of the helices will be improved, while the thermal conduction will increase due to larger cross-sections. Consequently, the latter should in turn decrease the local temperatures and increase the effective precursor population at the growth front. Together with the reduced current density for defocused electron beams, the efficiency is likely to increase, solving another problem of 3D-FEBID. As the beam diameter can be precisely controlled in modern instrumentation, this approach inherently provides tunability of branch widths, which would expand the flexibility of 3D-FEBID. The implications of such an approach on the co-deposit behavior beneath the intended structures is an open issue, which needs to be studied as well.

With this motivation in mind, the following work is intended to show the applicability of a deliberately defocused electron beam as a further process parameter for 3D-FEBID. To begin with, the effect of a defocused electron beam on the deposition of diving boards is shown and discussed. The gained knowledge is then used for the deposition of helices, which is discussed subsequently.

## 2. Part I—Diving Board Investigation

The introduction of a defocus method to an electron beam essentially increases the diameter of the electron beam. Since defocus and beam diameter are connected via the beam convergence angle—which is an instrumentation related size—we here specify the beam blur (added diameter to the in-focus diameter of the beam) instead. The increase in the cross-sectional area of the beam results in a decreased current density. Due to the quadratic dependence of the cross-sectional area on the beam diameter, the area increases quickly with increasing beam diameter. In turn, current density significantly decreases upon introduction of a beam blur (Supplementary Materials, Figure S1).

To investigate the effects of a blurred electron beam on 3D-FEBID, initial experiments were carried out employing diving boards (DB) as a test structure. DBs consist of a vertical pillar acting as a pedestal to minimize substrate related influences, followed by an inclined segment. Information about the growth dynamics can be gained from the investigation of the inclined segment, as done in the past [4,13,14,23,24]. The segment inclination ζ is defined as the positive angle between the segment with relation to the substrate plane. This geometry is used as calibration structure for CAD assisted FEBID [24] as segment inclination is a function of patterning velocity (PV) at in-focus conditions. Arrays of DBs were fabricated with target pillar heights of 800 nm and horizontally projected segment lengths of 3000 nm at dwell times (DT) ranging from 5 to 50 ms, with blur values in the range of 0 to 300 nm. For a constant point pitch (PoP) of 1 nm, the DTs translate to PVs ranging from 200 down to 20 nm·s^−1^. The actual beam diameter for our instrumentation is listed in the Supporting Information (Table S1). As the intention of this study includes the co-deposit formation as well, we decided to use low primary electron energies of 5 keV to minimize contributions from transmitting electrons. To provide comparability to previous studies, the beam current was fixed to 28 pA.

**Figure 1:**
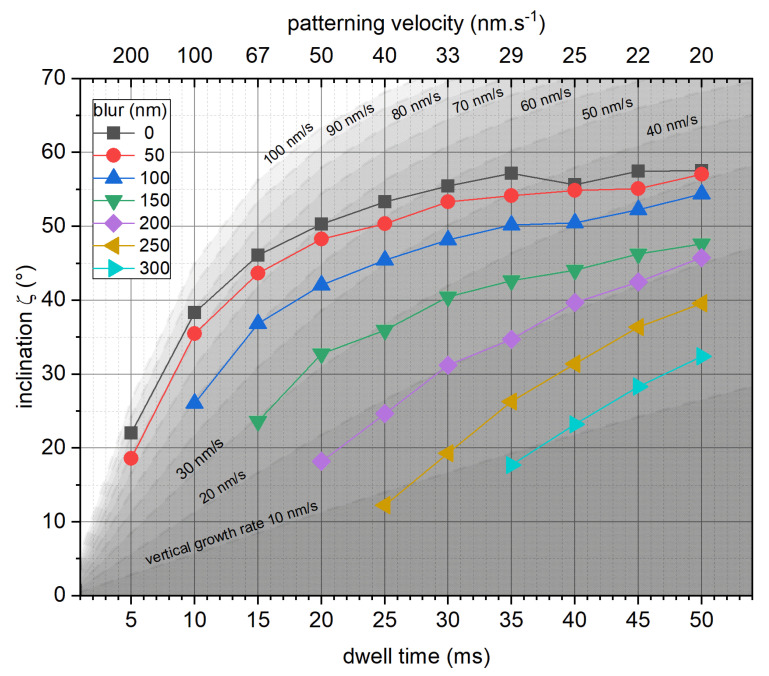
Implications of blurred electron beams on inclination ζ during 3D- focused electron beam induced deposition (FEBID). ζ is plotted as function of dwell time/patterning velocity for in-focus conditions and blurred electron beams at 5 keV/28 pA. The shaded areas indicate constant vertical growth rates (see main text).

Figure 1 assembles the average inclination angles ζ (please see Methods for further specification) independent of applied DTs, where the graphs are grouped w.r.t applied blur. Please note, this graph and the following graphs only include results of DBs that could successfully grow over a horizontal projected distance of at least 800 nm. The black squares represent the inclination angle for in-focus conditions, which increases with higher DTs, up to a saturation of around 60°, in agreement with previous findings [23]. The introduction of a blurred electron beam results in reduced segment inclination, enabling fabrication of less inclined segments also for higher DTs. The differently shaded background illustrates constant vertical growth rates for guidance (zVGR = PV·tan (ζ)). When relating the experimental data to such regions, it becomes evident that higher DTs lead to decreasing zVGRs up to a 150 nm beam blur. Even broader beams, however, show a trend inversion and lead to increasing zVGRs, which clearly indicates a regime shift. This is a very important detail, which will be discussed in more detail below.

**Figure 2:**
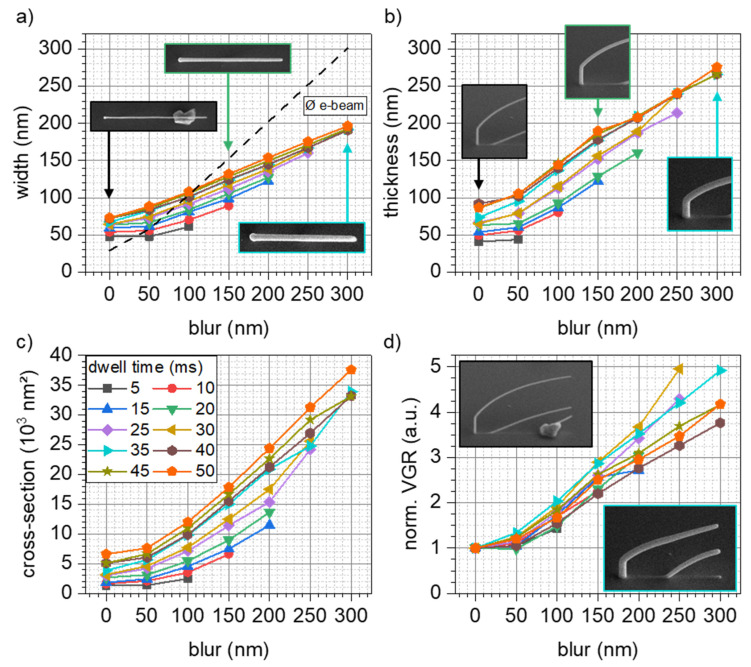
Implication of beam blurs on (**a**) segment widths, (**b**) thicknesses-, (**c**) cross-sectional areas and (**d**) relative efficiencies. All data are grouped by different DTs (see legend in c). While the dashed line in (**a**) indicates the calculated beam diameter by the instrument, the insets in (**a**,**b**) give directly comparable top and tilted SEM images, respectively. Volume growth rates were always normalized to in-focus conditions (blur = 0) to show the efficiency boost aside of absolute volume growth rates.

In contrast to the segments’ inclination, both segment-width and -thickness (measured perpendicular to the segment axis) increase with blur. Figure 2a shows the width in dependence of applied beam blur, grouped with respect to the applied DT. For in-focus settings, segment widths are between 50 and 75 nm. With increasing blur, the widths monotonically increase up to ~200 nm for the highest beam blurs of 300 nm, which means an increase by a factor of 2.6. The insets in Figure 2a show selected in-scale top view SEM graphs of DBs highlighting the width tunability. To avoid inclination dependent width convolutions, DBs presented in the inset are of comparable inclination. The dashed line in Figure 2a indicates the actual beam diameter. As is evident, the beam diameter is smaller than the segments for in-focus conditions. For higher blur values, the situation reverses, and the segment widths are below the beam diameter, which means that a fraction of primary electrons within the low intensity beam tails never impinge on the deposited structure. Segment thicknesses against blur are plotted in Figure 2b, again, grouped w.r.t applied DTs. At the in-focus conditions, segment thickness ranges between 40 and 90 nm, while the introduction of the beam blur increases the thickness by a factor of around three up to 275 nm (50 ms DT, see legend in Figure 2c). As a consequence of increasing widths and thicknesses, cross-sectional areas (elliptically modelled in agreement with our previous work [3]) increase as well, as summarized in Figure 2c. This -quantity is of particular interest for many application related properties. The mechanical load capacity and section modulus as well as electric or thermal properties are directly connected to the cross-sectional area. Across the applied settings, cross-sectional areas increased by almost one order of magnitude (~8.7 for DT 35 ms). The aforementioned gain in structural dimensions is solely down to increasing beam diameters, while the patterning velocity was kept constant in all experiments. This, in turn, means that the volume deposited per time and by that the efficiency is increased. To compare the volume growth rate increases across the applied beam parameters (blur and DT), Figure 2d shows the relative increase, normalized by the values at in-focus conditions. As evident, the efficiency is increased up to a factor of five, just by introducing the beam blur at constant beam currents and identical total exposure times for the same DT settings.

**Figure 3:**
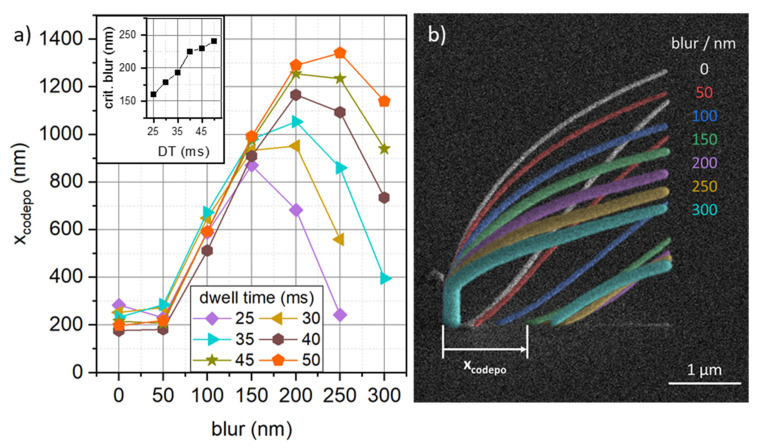
Horizontal distance upon formation of a co-deposit (X_codepo_) in dependency of beam blurs for different dwell times (DTs) (**a**) together with a superimposed, in-scale SEM image (**b**), which directly shows the delayed co-deposit by the introduction of beam blurs (grouped by color). The inset in (**a**) gives the DT dependent maxima of X_codepo_.

The application of a defocused electron beam also has an impact on the occurrence of co-deposition. In our experiments, every DT–blur combination resulted in the formation of co-depositions, as expected. In Figure 3, the horizontal distance, upon occurrence of co-deposition (x_codepo_, see Figure 3b), is plotted against applied beam blur for different DTs (see legend in a). For in-focus conditions and 50 nm blur, co-deposits form at a horizontal distance of 175–300 nm. Further increases in beam blur drastically increase the distance upon formation of a co-deposit, until a DT dependent maximum is reached (see inset in Figure 3a). For the longest DTs of 50 ms, co-deposition can be delayed to a horizontal distance of more than 1.300 nm at 250 nm blur, which is an increase by a factor of 6.8.

The aim of this preliminary investigation was to evaluate the implications of a blurred electron beam during 3D-FEBID. The presented experimental findings show that the introduction of a blurred electron beam affects segment inclination, width, thickness, cross-sectional areas and, in particular, the occurrence of co-deposition. In the following, we discuss the implications of blurring on the segment geometry and the co-deposit in more detail. 

**Figure 4:**
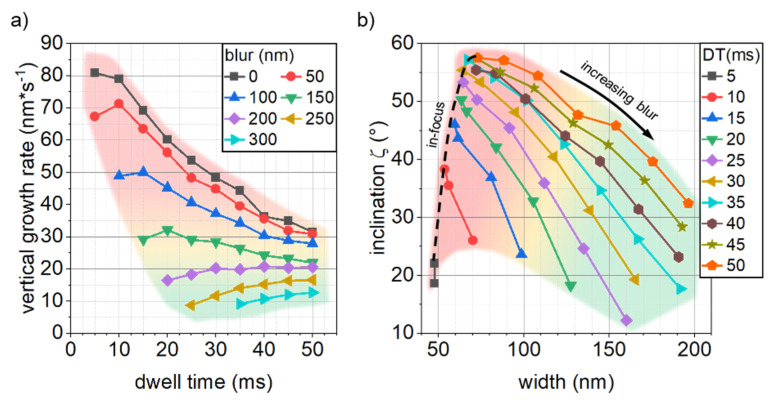
(**a**) vertical growth rate vs. DT for different beam blurs. The graph illustrates the working regime shift from molecule limited regime (MLR) (in-focus, blur < 200 nm, shaded red) towards more electron limited regime (ELR) (blur > = 200 nm, shaded green) conditions for increasing blur values. (**b**) demonstrates the enhanced tunability. While the dashed line illustrates one-dimensional, intrinsic width/inclination behavior, the introduction of a beam blur significantly expands the addressable width/inclination range. The color shading follows the code in (**a**), where green indicates the most balanced range for highest stability and efficiency.

The results for segment inclination, width and thickness and the related values cross-sectional area and volume growth rate strongly indicate a shift in the working regime upon application of a blurred electron beam. The working regime describes the relation between locally available precursor molecules and electron species present to participate in a dissociation event [25]. The electron limited regime (ELR) is characterized by a surplus of precursor molecules at the deposition site and a volume growth rate governed by the number of available electron species. In the opposite case, a lack of precursor molecules is due to insufficient replenishment limits the volume growth rate, which is called the molecule limited regime (MLR). As already mentioned, the experimental data for segment inclination displayed in Figure 1 show a trend towards lower vertical growth rates (dashed lines) for increased DTs for in-focus conditions up to a beam blur of 150 nm. For a more detailed discussion of this behavior, Figure 4a shows the experimentally obtained vertical growth rates zVGR against DT with graphs grouped to the applied beam blurs. In-focus conditions (black squares) show a steady decrease in vertical growth rate for increasing DTs, suggesting a decreasing precursor coverage characteristic for more MLR conditions. This behavior was also reported in [23]. Introducing a beam blur and thus reducing the current density, results in a lower vertical growth rate, in addition to the maxima in the vertical growth rate being shifted towards longer DTs. The increasing growth rate, present for blur values ≥50 nm, can be attributed to an inclination angle dependent volume growth rate due to increased pathlengths for electrons in steeper inclined segments [3,23]. At a blur of 200 nm (purple diamonds) and above the vertical growth rate shows no distinct maxima. Instead, vertical growth rates increase for larger DTs, suggesting a stable precursor coverage characteristic in more ELR conditions. Introduction of a blurred electron beam, and thus, reduced current density, result in a shift of the growth regime from MLR towards ELR conditions. The increased segment dimensions can therefore be attributed to the increased growth efficiency due to shifted working regimes. This regime shift is indicated in Figure 4a by different color shadings with red and green meaning MLR and ELR, respectively. The data reported here for blur values of 200 nm and above are in good qualitative agreement with vertical growth rates obtained for 5 keV and a beam current of 5 pA [23]. This means, that by applying a deliberate blur, the fabrication process can entirely be altered, as obtained for two different beam setups.

From a practical point of view, the introduction of a blurred electron beam allows for exquisite control over the final geometry of a deposit, as illustrated in Figure 4b, where segment inclination is plotted versus segment width. Please note, that the graphs are grouped w.r.t applied dwell times in contrast to Figure 4a. For in-focus conditions, segment inclination is a sole function of patterning velocities. Due to the angle dependence of growth rates, segment widths are tied to segment inclination and cannot be tuned unless a different beam setup is employed (dashed line in Figure 4b). Increasing the beam diameter via blurring allows for the deposition of segments with desired width and inclinations over a wide range. This is of essential importance for applications with a demand on cross-sectional areas, e.g., mechanical properties as well as properties governing the transport of heat or electricity. An alternative route to achieve the here presented fabrication flexibility, could be the application of advanced patterning approaches. However, such an approach requires upfront modelling and simulation of process dynamics to account for various effects influencing growth, e.g., proximity effects. Hence, the here presented approach is a direct way to tune segment dimensions during 3D-FEBID, while simulation assisted strategies will be elaborated in near future.

Both experiments [15] and simulations [17], have suggested, that transmitting electrons are involved in the co-deposit formation. As shown in Figure 3, the occurrence of co-deposition is delayed upon blurring the electron beam. Exceeding a critical blur value (maxima in a) then leads, again, to earlier co-deposit formation. The initial increase in distance upon formation of a co-deposit might be attributed to the increase in segment dimensions by the increasing blur, in particular the segment thickness. The penetration depths for 5 keV electrons in the relevant PtC_X_ materials is in the range of 200 to 300 nm [3]. Together with the experimentally determined results (Figure 2b), this means that—depending on the inclination angle—a fraction of electrons can still penetrate the structure. This, however, is in contrast with the observation of the maxima in Figure 3a, which means that although thicknesses are monotonically increasing, co-deposits occur again at shorter distances. This allows for the conclusion to be drawn, that transmitted electrons are not the only mechanism responsible for the formation of a co-deposit.

For a deeper look into the reasons behind the formation of co-deposition, we start the discussion with Figure 3a and, in particular, with the observation that the maxima are DT and blur dependent. Rearranging the co-deposit distances as a function of the inclination angle does not show a sole angle dependency but instead, blur dependent maxima (Supplementary Materials 2, Figure S2). In a follow up step, we evaluated whether the unexpected co-deposit behavior depends on the working regime by rearranging them as function of the applied dose. That approach reveals a collective maximum between 2500 and 5000 µC·cm^−2^, as shown in Figure S3 in the Supplementary Materials. This decouples the formation of the co-deposit from geometrical cross-sectional dimensions but maintains the inclination angle dependency with the dose. Before a first interpretation can be given, another detail was identified during high-resolution SEM inspection. The growth fronts of co-deposits are always situated ahead of the actual segment, as shown in Supplementary Materials 2, Figure S4. This strongly suggests that the co-deposit (aside of transmission contributions) is formed by a fraction of electrons, that miss the front apex. As a consistency check, we evaluated the horizontal distance between original and co-deposit apexes independent of blur, finding a linear trend (Supplementary Materials 2, Figure S4). Hence, we propose the increasing and eventually decreasing co-deposit distance to originate from different volumetric growths in forward direction, where increased volume leads to an increasing co-deposit distance and vice versa. Very similar processes have been observed before for focused ion beam induced deposition (FIBID), which became particularly evident for very small and even negative inclination angles (equivalent to low doses) [26,27]. The responsible mechanism is explained by two competing processes. At very low doses, the spatial growth including the front volume is small, which leads to unstable growth and small co-deposit distances [23]. Increasing the dose results in more electrons and generally higher volumetric growth in all directions. In turn, a smaller fraction of electrons can pass ahead of the apex, which delays the formation of a co-deposit. While this trend is expected to be increasing and eventually saturating [3], EBIH has to be taken into account as well. Higher doses mean higher angles, longer segments, higher thermal resistances and higher temperatures in the beam impact region. As consequence, the effective available number of precursor molecules decays, which reverses the volumetric growth trend. As this affects the front volume as well, an increasing fraction of electrons can pass ahead of the segment apex, which again reduces the co-deposit distance. That explains the experimentally observed behavior of the co-deposit distance, which increases, then peaks and eventually decreases again as a function of the applied dose, regardless of the applied blur and related cross-sectional areas. However, to fully understand the co-deposit formation, and in particular the role of transmitting electrons, further simulation/experimental studies are needed in the future. As an intermediate summary of part I, we have demonstrated the applicability of a deliberate beam blur as an additional, highly useful parameter for 3D-FEBID. The main advantage is the expanded flexibility, as cross-sectional dimensions can precisely be adapted to the final application requirements. At the same time, deposition efficiencies strongly increase, while unwanted co-deposit formation can be delayed up to a certain extent due to fundamental coverage limitations, driven by EBIH. With this tool in hand, we now turn back to the initially mentioned fabrication of helices.

## 3. Part II-Helix Fabrication

**Figure 5:**
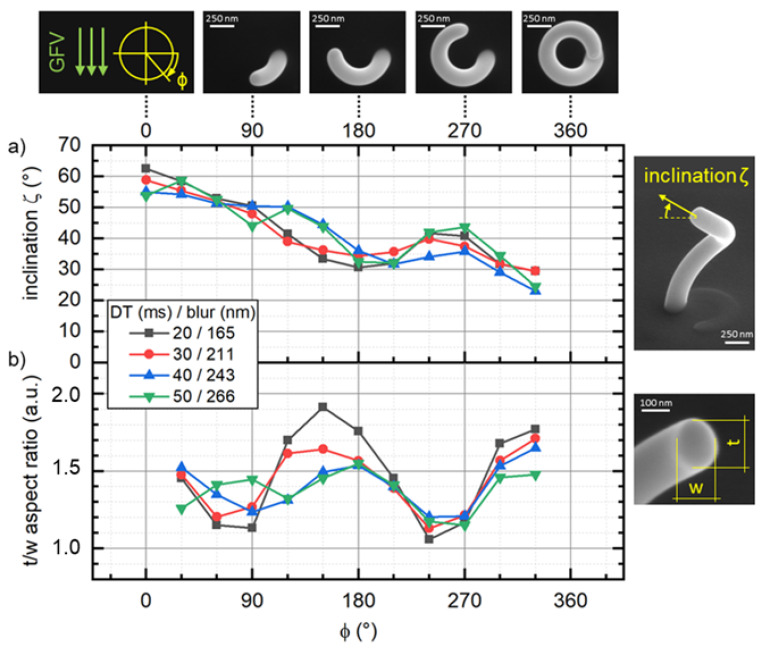
The evolution of inclination angles (**a**) and aspect-ratios (**b**) along the growth of 500 nm wide single turn helices, specified by the azimuthal angle ϕ. The top row shows SEM top view images to illustrate the successive clockwise growth of a helix together with the direction of the gas flux vector (GFV). Right column indicates the inclination angle ζ, the widths w and the thicknesses t. Both azimuthally resolved information are given for different blur values, where DT are adapted (see legend) to achieve a target inclination angle of 45°.

The controlled and stable fabrication of open helices is a challenging task, as it actually is a re-directed single wire, which has been demonstrated to be problematic, once their lengths approach the micron range [27]. Additionally, varying angles between the directed gas flux and the growth direction arise, which can impact the spatial precision [10,21,28]. As a starting point, helices with a diameter of 500 nm were deposited at a range of constant DTs, i.e., patterning velocities at adjusted blur values aimed to result in a target inclination of 45°. For deposition, the electron beam was moved clockwise, resulting in a left-handed helix. The top row in Figure 5 shows the gas flux vector (GFV, green arrows) together with four different growth states, visualized by top view SEM images. As is evident, the growth always started at the three o’clock position (azimuth angle ϕ=0°), which means that growth direction (GD) and GFV are parallel at the beginning. After full deposition, the ready structures were imaged from top to access the width of the structure in dependence of ϕ. To measure the vertical e-path (segment dimension parallel to beam axis) of the helix as function of ϕ, tilted SEM graphs (52°) were taken, while the stage was rotated in steps of 30° azimuth for a full rotation. Helix inclination ζ (see SEM inset at the right) was then calculated from the measured height increments and the segment thickness t was determined from the e-path and the calculated inclination (t=e−path×cos(ζ)). The cross-section was assumed to be elliptic, as found in a recent study [3], which allows calculation of the cross-sectional areas as a function of w, t and ζ. To assess the deposited volume, segment length was determined from the incremental height increase and the projected arc length followed r×∆ϕ, with r being the helix radius and ∆ϕ = 30°. Volume growth rate were obtained by dividing the calculated volume for the azimuth increment of 30° by the projected arc length. Please note, that volume calculations assume a helix of constant curvature and torsion [29], which entail certain errors in absolute volume growth rates and efficiencies. The deposited coils show a total height of (1510 ± 50) nm, which results in an average inclination of 45°. Please find the graph illustrating the height as a function of the azimuth angle in Figure S5 (Supplementary Materials 3). The azimuthally resolved inclination angle of the helical structures is plotted in Figure 5a. Starting at ζ take-off angles in the range of 53–62°, the inclination runs through a minimum of around 30–35° at an azimuth angle ϕ of 180–210° followed by a slight increase to 35–45° at an azimuth angle of 270°, followed by another decrease. Please note, that inclination could not be obtained for an azimuth angle of 360° reliably, as the growth front at this angle did not allow accurate measurements of the actual e-path length.

The take-off angles clearly exceed the targeted inclination of 40°, which, to our understanding, is reasoned by close proximity to the substrate, where substrate related diffusion has a strong impact [3]. For the following half-turn, inclination is steadily decreasing until the GD is antiparallel to the GFV (180° azimuthal), where the trend first reverses up to 270° and then decays again. As EBIH would lead to a monotonic decay along the full turn, we attribute the quarterly modulation to the varying angles between GFV and GD, where patterning towards the gas flux is obviously beneficial. Both together could then explain the observed behavior by a combination of angle modulation with a continuous decay. Aside of the inclination angle, the cross-sectional shape evolution along the full turn is of interest as well. As shown in Figure S6 (Supplementary Materials 3), w and t also exhibit non-monotonic dependencies on azimuthal angles ϕ. While t reveals two local minima at ϕ ranges of 90–120° and 240–270°, wire width w has a local minimum around 135° azimuth angle (details can be found in Supplementary Materials 3). Figure 5b shows the aspect-ratio (t/w), which reflects the cross-section modulation along the wire. As evident, there are clear minima (90°/270°) and maxima (180°/360°), which become less intense with increasing beam blur (see legend). The latter means that vertical and lateral growth becomes more balanced, which is an indication for more balanced precursor-to-electron ratios. This agrees with the diving board findings, where the blur is suggested to shift the local working regime more towards more ELR conditions.

**Figure 6:**
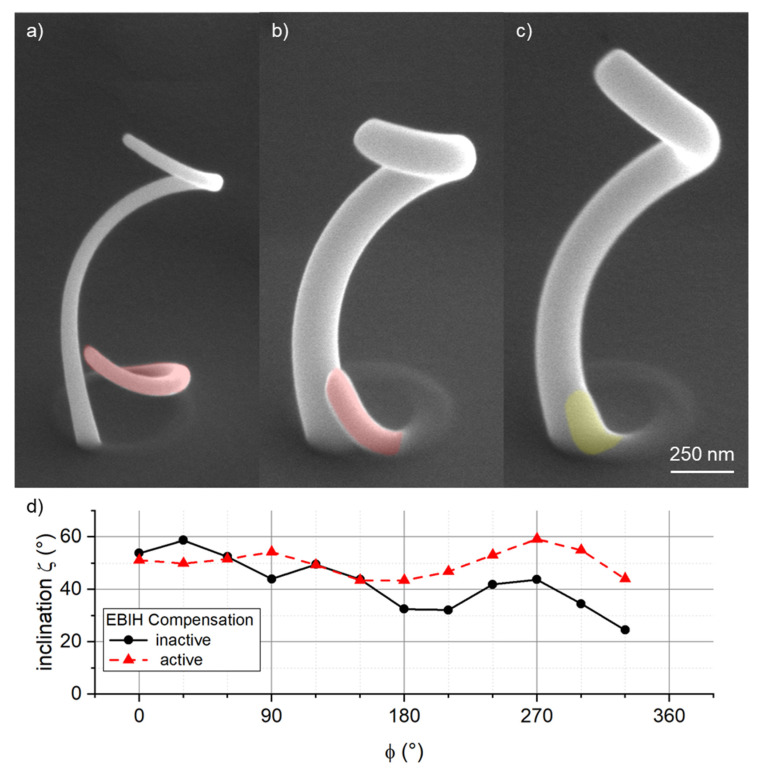
The impact of beam blurs on 3D helices. In contrast to in-focus conditions (**a**), 0 nm blur, 7 ms DT, a blurred electron beam widens the segments cross-section as intended and delays the occurrence of co-deposition (shaded red) (**b**), 266 nm blur, 50 ms DT. The azimuthally resolved inclination angle of the latter is shown in (**d**) by black circles, which reveal a general decay, which is also qualitatively evident in the SEM image in (**b**) (compressed upper part). That effect can be related to electron beam induced heating (see text) and can be compensated, as evident by the red triangles in (**d**). The corresponding SEM image in (**c**) clearly confirms the improved quality of the helix and furthermore reveals no lift-off co-deposit underneath (shaded yellow, compare to (**b**). Please note the always evident, thin ring underneath the segments, which suggests transmitting electrons in agreement with the literature.

Co-deposition occurred for all fabricated helices, which is no surprise considering the results from the diving board experiments. The maximum distance without co-deposition was around 1350 nm (Figure 3), whereas the projected circumference of the deposited helices is more than 1500 nm. Figure 6 shows a comparison of helices where (a) was deposited with a focused electron beam at a DT of 7 ms and (b) was fabricated applying a blur of 266 nm and a DT of 50 ms. At in-focus conditions, co-deposition (shaded red) starts to lift-off at an azimuth angle of around 165° which equals a distance upon co-deposit lift-off of around 720 nm. Using the blurred beam resulted in a delayed occurrence of co-deposition at around 310° azimuth. This equals to a length on the circumference of around 1350 nm, the same value obtained as for the DBs. It should be noted though, that the present structures were fabricated at a constant DT, i.e., constant patterning velocity and EBIH still affects the growth rate, considering the decrease in inclination (discussed above) and structural dimensions. To further delay co-deposition, a simple method was applied to compensate for the effect of EBIH, i.e., the steadily decreasing precursor coverage at the deposition site. Basically, the patterning velocity must be reduced such that the reduced precursor coverage ensures stable and constant growth rates [17,27,30]. A number of elaborate strategies have been proposed already, which usually require calibration structures or simulation results as an input. Here, our aim was not to develop a new compensation approach, yet we could also not take a present compensation model without the effort to calibrate the underlying model to the fabrication using a blurred electron beam. Hence, the applied approach uses a cubic polynomial to increase the DT in dependence of the deposition steps, according to $DT\left(x\right)=DT_{0}+a\times x^{3}$ with x representing the number of deposition steps and a the compensation factor. For the present case, with a constant PoP of 1 nm, x also represents the horizontal distance covered. For a, factors on the range of 10^−8^–10^−9^ ms∙nm^-3^ have been applied. The structure displayed in Figure 6c was deposited at the same parameters as (b) but in this case the presented DT-compensation was active with compensation factor $a=1.4\times 10^{-8}\;\mathrm{ms}\times {\mathrm{nm}}^{-3}$. As evident from the SEM graph, co-deposition could be almost completely avoided, with a slight bump still visible at the base of the segment (shaded yellow). In Figure 6d, the inclination is plotted against the azimuth angle for the structures depicted in (b) and (c). Apparently, the applied compensation approach (red triangles) is able to remove the monotonic decrease by EBIH (compare to black circles), while the four-quadrant like inclination modulation still remains. This is a further indication that the latter is indeed a directional gas flux effect, with the angle between the GD and the GFV being the decisive element. Depending on the target applications, further adaption could be needed to stabilize both inclination and aspect-ratios along the azimuth angle.

## 4. Conclusions

The applicability of deliberate beam blur as an additional process parameter during 3D-FEBID was investigated on a wide range of parameters. Blurring the electron beam, which effectively reduces the current density, can shift the working regime towards much more stable conditions with efficiency boosts of up to a factor of five. At the same time, the mostly unwanted formation of co-deposits beneath the intended structures can be strongly delayed into the micron range. The essential feature of blurred beams, however, is the precise tunability of nanowire dimensions with cross-section increases of up to one order of magnitude. Consequently, the approach is faster, more stable, less prone to parasitic growth and allows for specific design adaptions to the target requirements (mechanical, thermally, electrically, optically). Together with the fact, that blurred beams are easy to apply from a technical point of view, this approach is an essential element to enhance the design flexibility of FEBID-based 3D-nanoprinting.

## 5. Methods

Deposition experiments were conducted at a Dual Beam microscope (Quanta 3D FEG, Field Electron and Ion Company, (FEI)) fitted with a standard gas injection system (GIS) by FEI. For all structures, trimethyl-(methylcyclopentadienyl) platinum (IV) (MeCpPt^(IV)^Me_3_, CAS: 94442-22-5) was used as the precursor gas. The GIS was positioned 100 µm above the substrate surface and at a radial distance of 100 µm to the beam center. The precursor was heated for at least 1 h at 45 °C prior to any deposition experiments. The GIS valve was opened at least 5 min before fabrication to achieve thermodynamic equilibrium w.r.t precursor adsorption. Base pressure inside the SEM was 5–7 × 10^−6^ mbar and increased to 0.9–1.3 × 10^−5^ mbar upon opening the GIS valve. For all experiments, the point pitch (PoP) was kept constant at 1 nm. Patterning was conducted using stream-files (diving board and circular pattern). All structures were deposited at an acceleration voltage of 5 keV with an aperture of 20 µm and spot size of 4.5 resulting in a beam current of 28 pA. For quantification of the segment’s projected height, inclination and segment length, tilted (52°) SEM images were used, whereas top-view images were used to record the segment’s width. Image analyses of SEM graphs were conducted using the software package FIJI [31] (which is an ImageJ package [32]) in conjunction with a python script. Structural dimensions (inclination angle, width, thickness etc.) were derived from the average between 250 nm and 750 nm horizontal projected length to exclude the less defined pillar-segment transition region and the impact region at the segment end.

## Supplementary Materials

The following are available online at https://www.mdpi.com/2072-666X/12/2/115/s1, Table S1: beam defocus to blur conversion, Figure S1: Beam current densities in blurred conditions, Figure S2: Co-deposit delay as function of inclination angle, Figure S3: Co-deposit delay as function of applied dose, Figure S4: Co-deposit ahead growth, Figure S5: Azimuthally resolved, nano-coil height growth, Figure S6: Azimuthally resolved width evolution during nano-coil growth, Figure S7: Azimuthally resolved thickness evolution during nano-coil growth, Figure S8: Azimuthally resolved cross-sectional evolution during nano-coil growth
