# Supplementary material for: Expanding 3D Nanoprinting Performance by Blurring the Electron Beam
Source: Micromachines (Basel). 2021 Jan 22;12(2):115. doi: 10.3390/mi12020115 (PMC7911092; doi:10.3390/mi12020115)
Supplement: Supplementary file 1 [file micromachines-12-00115-s001.pdf]

micromachines

Supplementary Materials: Expanding 3D Nanoprinting Performance by Blurring the Electron Beam

Lukar Matthias Seewald, Robert Winkler, Gerald Kothleitner and Harald Plank

Supplementary Material 1: Beam Diameter and Current Density Upon Blurring

Blurring the electron beam increases the beam diameter of impinging primary electrons. Table S1 lists beam blur values and resulting beam diameters for the chosen setup of 5 keV / 28 pA. The 3σ beam diameters were taken from the patterning metadata file (ptf), provided by Microscope software (FEI Company, Netherlands). While Gaussian like intensity profiles [1] apply for in-focus conditions, defocused beams often deviate from that distribution and approach a more rectangular intensity distribution, also observed and reported for our instrumentation [2]. That enables an estimation of the average current density in dependency on the beam blur. As evident in Figure S1, the current densities (logarithmically scaled) reveal a rapid decrease with the beam blur (see also the linear scaled plot as inset). Over the applied range of additional beam blur (0–300 nm), the beam diameter is increased by a factor of 10, thus the averaged current density decreases by two orders of magnitude.

Table S1. Beam blur values and according beam diameter.

blur/nm	$Ø_{beam}$/nm
0	29.2
50	57.9
100	104.2
150	152.8
200	202.1
250	251.7
300	301.4

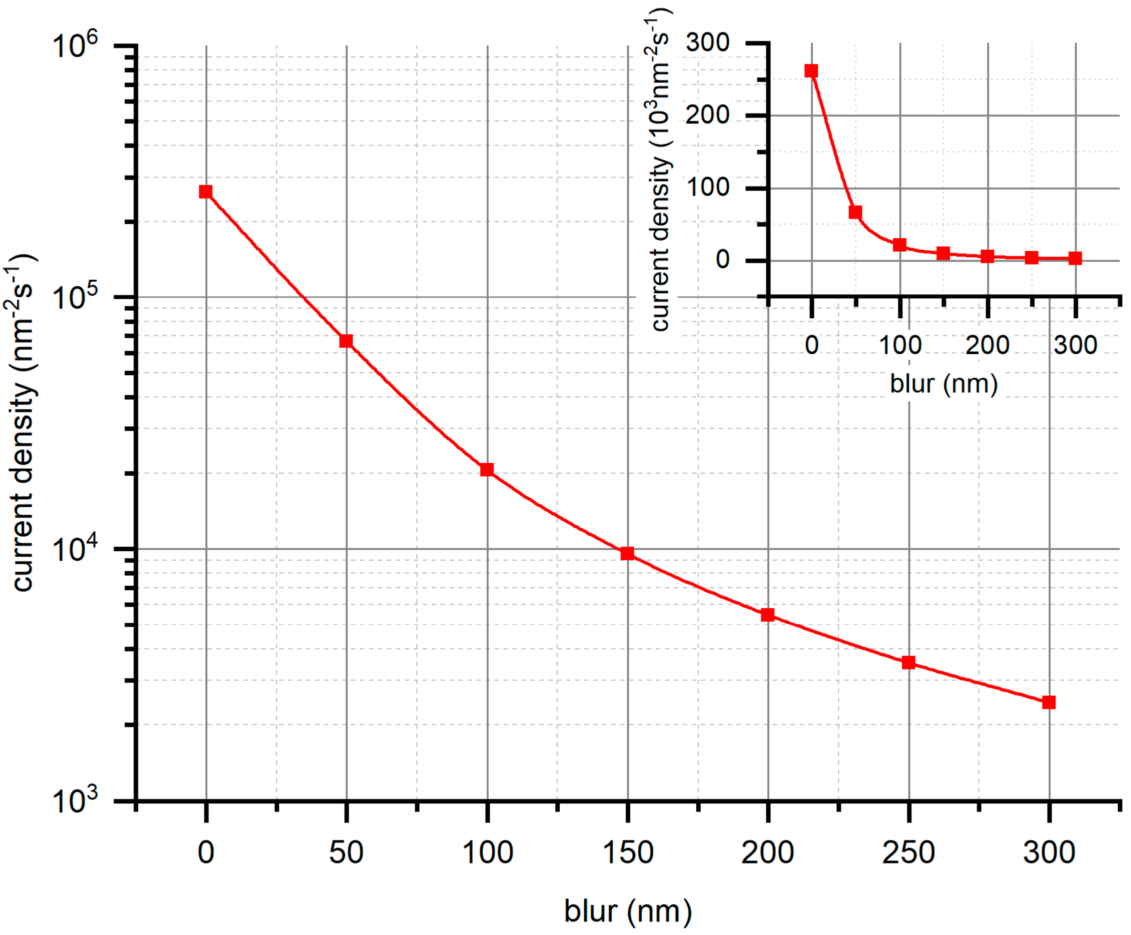

Figure S1. Decrease in current density upon introduction of a beam blur. While the main plot has a logarithmic ordinate, the inset shows the real situation, where the fast decrease is clearly evident.

Supplementary Material 2: Co-Deposition

Figure 3 in the main manuscript illustrates the delay of co-deposition upon blurring the electron beam and to derive process parameters, x_{codepo} was plotted against applied blur. Figure S2 plots the distance upon codeposition x_{codepo} against inclination ζ. The x-axis is reversed to represent the data with increasing beam blur from left to right. The graph does not show a sole angle dependency but still blur dependent maxima.

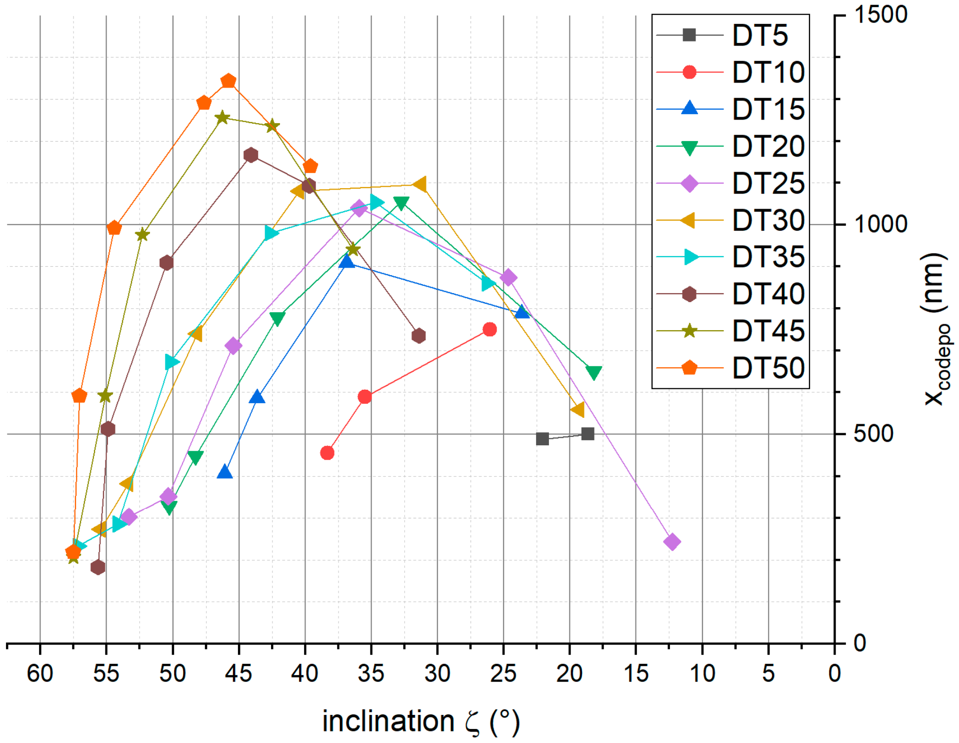

Figure S2. Distance upon co-deposition x_{codepo} plotted against inclination ζ. Please note the reversed x-axis which was chosen to represent the data with increasing beam blur from left to right.

Figure S3 plots x_{codepo} against the actual dose to normalize the data w.r.t the applied DT/PV, please mind the reversed logarithmic scale of the x-axis. The actual dose was calculated as the averaged dose over the entire beam area. Apparently, the maximum of x_{codepo} is reached on a narrow range of doses compared to the doses for a focused beam, roughly ranging from 2500 to 5000 μC·cm^{-2}. Slightly lower doses result in significantly smaller x_{codepo}.

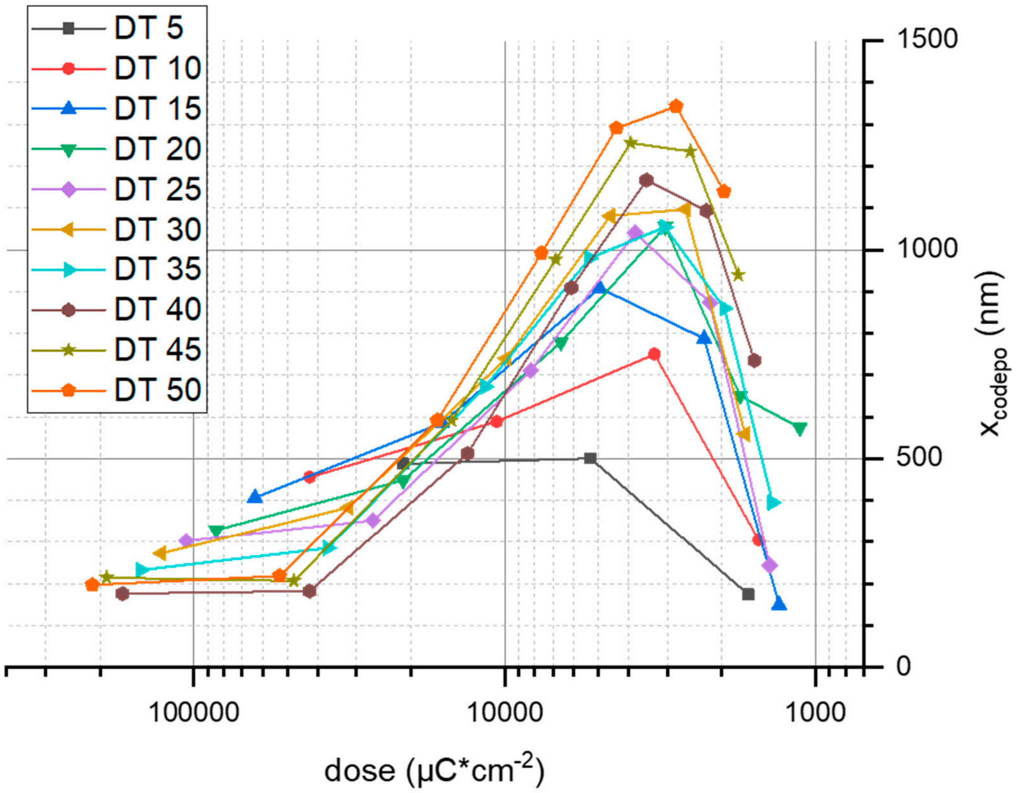

Figure S3. Plotting x_{codepo} against actual dose shows that the maximum in x_{codepo} is reached in a narrow dose range (2500 - 5000 µC·cm^{-2}). For lower doses x_{codepo} decreases significantly. Please note the reversed logarithmic x-axis, which was chosen to represent data with increasing blur from left to right.

Further insights into the growth of co-deposits can be gained by investigating the morphology of grown structures. Of particular interest here is the distance between the desired segment's growth front and the one of the co-deposit. Figure S4 plots tilted SEM graphs of straight segments deposited at a DT of 45 ms. Those segments are of considerable thickness, thus bending upon irradiation during imaging should be comparably small. The applied beam blur is specified in the top left of each graph. As is evident from the images, the distance between the two growth fronts increases with increasing beam blur / decreasing dose. Apparently, this behavior shows a linear trend with respect to applied beam blur.

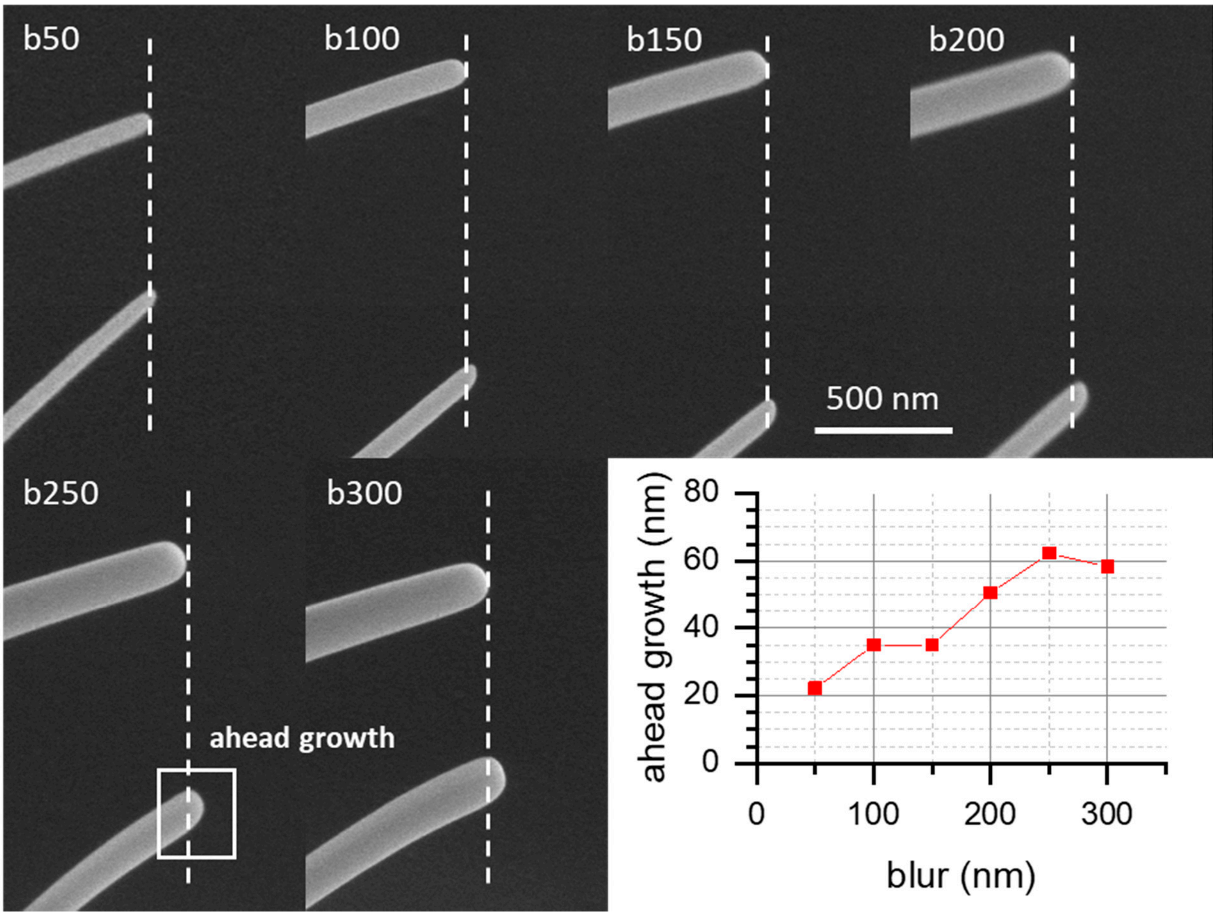

Figure S4. Defocus dependent ahead growth of co-deposits. The SEM images show always the intended original segments (upper parts) together with the co-deposit (lower parts), grown at constant DTs of 45 ms but increasing beam blur. The quantification is summarized bottom right, which clearly reveals an increasing ahead growth for broader beams, which supports the hypothesis of co-deposit formation due to electrons never interacting with the intended segment.

Supplementary Material 3: Geometry of helices

To complement the main manuscript, we here show additional analyses. First, Figure S5 shows the height coordinate of the neutral axis as a function of azimuth angle as indicated in the tilted SEM image. As a guide to the eye, the dashed and dotted lines represent a constant inclination of 45° and 50°, respectively. As evident, differently blurred beams (with adapted DTs to be in the correct inclination angle range) show a very similar behavior with final heights in a band of less than 100 nm, which demonstrates applicability, predictability and reliability of blur as additional 3D-FEBID process parameter.

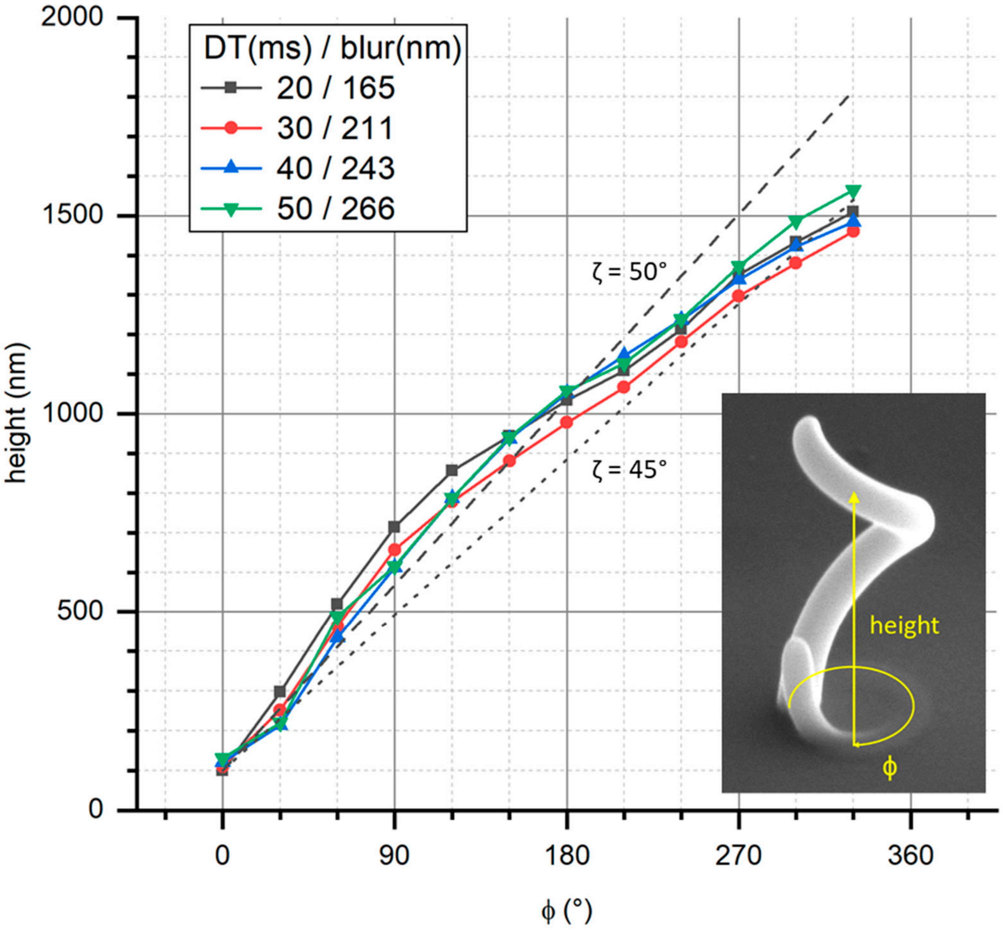

Figure S5. Height coordinate as a function of azimuth angle. Inset shows tilted SEM graph indicating the height coordinate. Dashed and dotted lines are a guide to the eye, representing 45° and 50°.

The helices' wire widths w are plotted in Figure S6. It was not possible to measure the width for an azimuth angle of 0° because the end of the helical wire partly blocked the sight towards the base of the helix. The curves are qualitatively similar with local minima around 135° azimuth, followed by local maxima peaking at an angle of 240° azimuth. Normalization to the first value reveals, for shorter DTs / smaller beam blur the relative change in width is larger and the subsequent increase in width is stronger, promoting the suggested regime shift for increasing blur values. The more molecule limited growth regime present for the shorter DT and smaller blur settings causes a stronger dependence on the angle between GFV and GD.

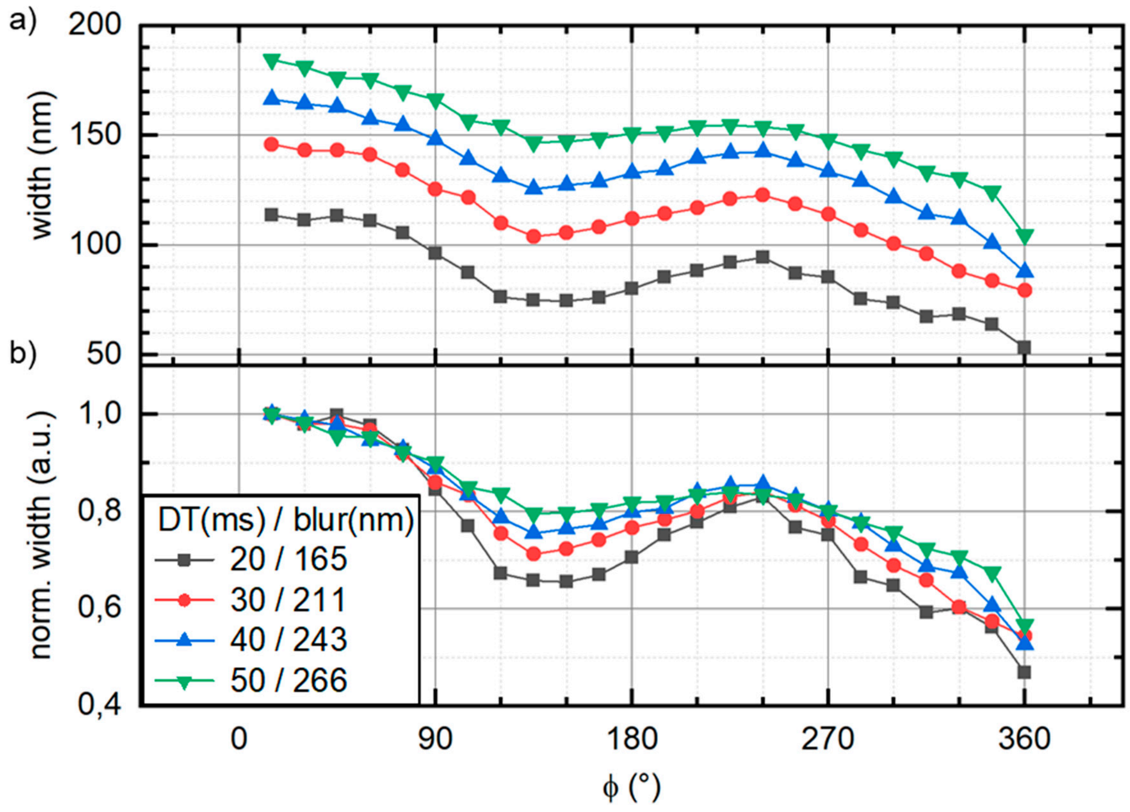

Figure S6. Widths in absolute (**a**) and normalized (**b**) representation (w.r.t the first value) plotted against azimuth angle.

The thickness of helical wires is computed as the product of e-path times the cosine of the according inclination (t = e-path·cos(ζ)) and plotted in Figure S7. The maximum thickness at azimuth angles of 30° can be attributed to the close proximity to the substrate with icreased diffusive replenishment and no impact of EBIH. Again, the resulting structures show similar trends with two local minima around 90° and 240–270° azimuth. Similar to the width results the thickness of the deposited structures varies less for larger beam blurs applied indicating more stable growth conditions.

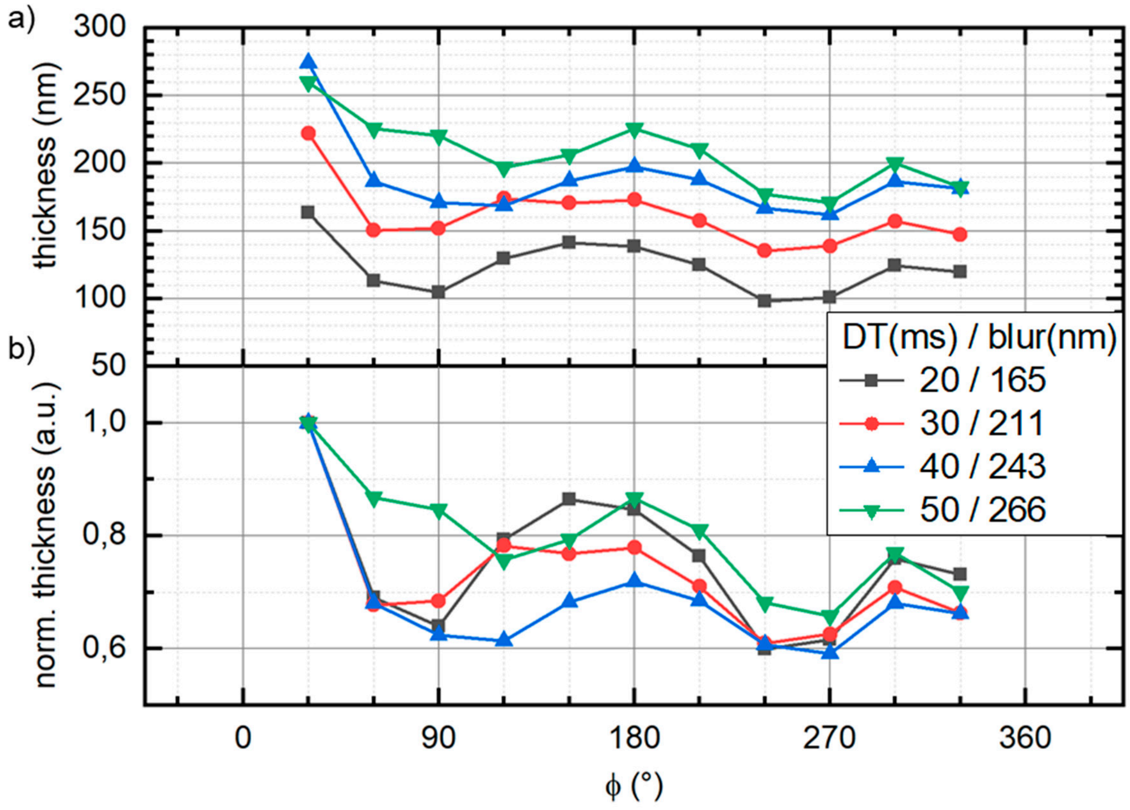

Figure S7. Thicknesses in absolute (**a**) and normalized (**b**) representation (w.r.t the first value) plotted against azimuth angle.

To illustrate the enhanced dimensional control enabled via blurring the electron beam, Figure S8 plots the cross-section against azimuth angle. While the inclination is almost identical (see Figure S4) the cross-section can be altered on a large range. This allows to tailor structural dimensions in accordance to functional requirements e.g in charge or heat transport.

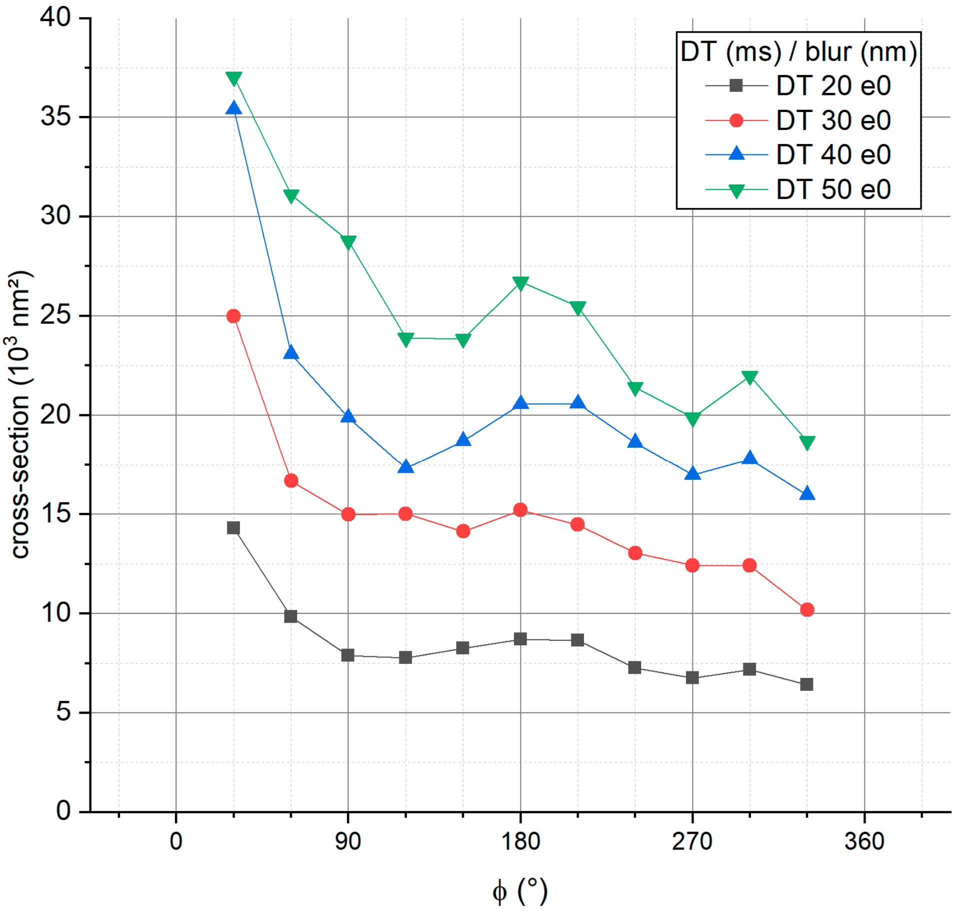

Figure S8. Cross-section plotted against azimuth angle illustrating the enhanced dimensional control. The cross-section can be altered for almost identical inclination.

References

1. Utke, I.; Moshkalev, S.; Russell, P. *Nanofabrication Using Focused Ion and Electron Beams*; 2012; ISBN 9780199734214.
2. Kuhness, D.; Gruber, A.; Winkler, R.; Sattelkow, J.; Fitzek, H.; Letofsky-Papst, I.; Kothleitner, G.; Plank, H.; Letofsky-Pabst, I.; Kothleitner, G.; et al. High-Fidelity 3D-Nanoprinting of Plasmonic Gold Nano-Antennas. *ACS Appl. Mater. Interfaces* **2020**, doi:10.1021/acsami.0c17030.
